# Supplementary material for: Reaching articular limits can negatively impact embodiment in virtual reality
Source: PLoS One. 2022 Mar 2;17(3):e0255554. doi: 10.1371/journal.pone.0255554 (PMC8890650; doi:10.1371/journal.pone.0255554)
Supplement: Attachment — Submitted filename: Response to Reviewers.pdf [file pone.0255554.s001.pdf]

Response to Reviewers

We would like to thank the reviewers for their helpful feedbacks. In order to reflect these modifications and ease the reading, the modifications in the paper have been done respecting these two rules:
_All the text planned to be removed have been ~~struck through horizontally.~~
_All the text added have been highlighted in yellow.
_All the modification are indicated with the corresponding number or letter of the reviewer comments. The numbers are associated to the comments of the reviewer 1 and the letters are associated to the comments of the reviewer 2.

Reviewer #1:

This paper reports an experiment where different configurations of the real and virtual arm in outstretched position are examined for their effects on body ownership of the virtual body. The experiment is well described and the statistical analysis is excellent. There are a few points that could improve the paper.

1) Although the submission form states that the data is fully available, I could not see where this was indicated in the paper - i.e., from where the data can be accessed.

The data can be found on this drive:

https://drive.google.com/drive/folders/1M9tgsQHYpSixnpDbRowvsHQ5cqhaB--r?usp=sharing

For the final publication a DOI toward a Zenodo account will be provided. Since once the Zenodo is created it cannot be deleted we are waiting the final version before creating the repository.

2) In 'Equipment and software' please give the weight of the head mounted display.

This information has been added into the paper.

3) In the next paragraph it is not clear whether it is only the virtual hand that holds the cylinder or whether subjects were also holding a cylinder. This seems to be cleared up later in the figure caption - that they were actually holding the cylinder. Was the cylinder tracked, or were transformations to the cylinder computed from transformations to the hand orientation and translation?

The transformations to the cylinder were computed from transformations to the hand orientation and translation. This was enough for the subjects to feel as if we were tracking the fingers since they were resting their fingers in the same position during the experiment. This information has been added earlier in the paper.

4) Some of the English reads a bit strangely and the paper should be thoroughly checked for this. For example, "All subjects have been through the two blocks" rather than "All subjects completed the two blocks".

"Although this question directly addresses only body ownership, it aims at informing on the impact of the articular limit on embodiment."
" ... at informing on ..." doesn't seem to be a correct formulation.
"subjects shall experience a strong sense of agency for the avatar's movements" in the discussion - the "shall" makes it seem like an imperative.

Thank you for all these corrections. We have added all these modifications and carefully read through all the paper.

5) Figure 5 - the questionnaire scores run from 0 to 1 but the vertical axes extend to 1.4. This is confusing and must be corrected. I think that all the significance *s above the bars need to be pruned, there are too many and the graph becomes difficult to read. Please only include the critical ones that relate directly to the hypotheses.

The figure 5 has been modified to show only the relevant information to ease its reading.

6) From the graph it is clear without any significance tests that the critical impact is associated with "Negative Distortion Fully Extended" and "Positive Distortion Fully Extended" which both result in an obvious reduction in body ownership compared to the others. All of the others, even though there may be some significant differences between them, show relatively high levels of body ownership. So "fully extended" with any kind of distortion leading to lower body ownership is the main finding. Although there are differences between the other conditions can the authors please mention also the effect sizes of these differences rather than only significance levels.

The effect size is computed using the scaled robust Cohen's standardized mean called ***dr*** in the text.

7) In the Results first paragraph I think it would be helpful to readers to first discuss the findings from the graphs in terms of what seems to be greater than what before rushing into discussion of significance levels without explaining what these mean. E.g., we can be told that "x is significantly different from y" without knowing whether this indicates whether x > y or y > x. So it may be preferable to give the latter information first, and then discuss the significance.

The results part has been modified to reflect the suggested presentation order of the information. Now it will be easier for the readers to understand what is > or < before knowing about the statistical results.

8) In the discussion it says that "subjects severely reject a discrepancy between their own body". But looking at the raw data 'severely reject' seems to be an over statement. It seems that only in the case of "fully extended" with any kind of distortion is there a clear drop in body ownership compared to the other conditions. All of the others are way above the 0.5 mark on the questionnaire scale.

The sentence has been modified to mitigate the interpretation coming from the observed results and reflect a more reasonable statement.

9) In the discussion "subjects might be more willing not to notice the distortion" - "willing" makes it seem like this is a conscious deliberate choice on the part of the subjects.

The meaning of the sentence has been changed to reflect an internal process.

Overall this is an excellent and useful contribution to the area of virtual embodiment, and will be important for researchers in this field.

Reviewer #2:

The manuscript is well written and the study design is well done. The results from this study can be helpful for conduction virtual reality experiments with healthy and clinical populations including embodied conditions. However, the authors should consider some aspects for improving the quality of the manuscript.

- Introduction section:

A) line 3: add a reference after SoE.
Suggestion for improving definitions of self-location, agency and body ownership:
Sense of Self-location: Self-location is a determinate volume in space where one feels to be located. Normally self-location and body-space coincide in the sense that one feels self-located inside a physical body (Lenggenhager, Mouthon, & Blanke, 2009).
Sense of Agency: The sense of agency refers to the sense of having ‘‘global motor control, including the subjective experience of action, control, intention, motor selection and the conscious experience of will’’ (Blanke & Metzinger, 2009, p. 7).
Sense of Ownership: Body ownership refers to one’s self-attribution of a body (Gallagher, 2000; Tsakiris, Prabhu, & Haggard, 2006).

Previous definitions have been replaced the one proposed.

B) Third pagraph: the authors say 'we identified a relatively frequent problem likely to interrupt the visuo-proprioceptive integration' ,where you identified this problem? Is there any study arguing this situation? If yes, please cite it.

We are not aware of prior studies discussing this context of visuo-proprioceptive integration disruption. We may make our sentence less formal by replacing "identified" by "observed".

C) End of the 4th paragraph: I agree with the impact of internal haptic feedback on movement perception in VR, however the authors should consider that if reserachers provide strong sense of agency making participants actively participate in the initiation of the virtual body movement or vistual body action, this may descrease or delete the mistmatch conflicts, and providing synchronous visuo-tactile correlations. See Kokkinara, E., Kilteni, K., Blom, K. J., & Slater, M. (2016). First person perspective of seated participants over a walking virtual body leads to illusory agency over the walking. Scientific reports, 6

This has been added to the paper with the corresponding refrences.

D) Participants section: not controlling the dominance laterality should be added as a limitation of the study. Even though the difuclty of the task, the joint receptors of the participants will be more sensitive, it should be a higher proprioceptive sense in the dominant upper limb compared to the non-dominant upper limb.

Indeed, this could be seen as a limitation and has been added in to the paper.

E) Discussion: the authors should consider that there are evidence showing that it is poosible to provide a distorted fedback of the virtual arm, such as an hyperextension of the upper limb, while providing synchronous multisensory correlations (e.g visuo-tactile feedback), and participants reported higher levels of wnership toward such distortion:
- Kilteni, K., Normand, J. M., Sanchez-Vives, M. V., & Slater, M. (2012). Extending body space in immersive virtual reality: a very long arm illusion. PloS one, 7(7), e40867.

Further, other studies showed that it is possible to provide body ownership illusions toward virtual bodies with distorted positions while providing synchronous visuo-tactile or visuo-motor correlations:

-Bergström, I., Kilteni, K., & Slater, M. (2016). First-person perspective virtual body posture influences stress: a virtual reality body ownership study. PloS one, 11(2), e0148060.

- Matamala-Gomez, M., Nierula, B., Donegan, T., Slater, M., & Sanchez-Vives, M. V. (2020). Manipulating the perceived shape and color of a virtual limb can modulate pain responses. Journal of clinical medicine, 9(2), 291

Indeed, movement distortion is not compatible with a good level of body ownership and corresponding refences have been added to support this idea.

F) Avatar representation section: Change the format about the take a home message. I suggest the authros to re-formulate a sentence as an statment.

The message has been changed according to this suggestion to reinforce the strength of our message.

G) Conclusion section can be shortened.

The discussion has been changed and the conclusion shortened.
